# Analytical performance and standardization of four HCV RNA assays in China: an evaluation of sensitivity, precision, and genotype inclusivity

**DOI:** 10.3389/fmicb.2025.1594410

**Published:** 2025-06-02

**Authors:** Hao Yan, Bing Xu, Qinyi Zhang, Lijun Zhang, Zhihan Fang, Huijun Zhang, Junhang Pan, Wenge Xing

**Affiliations:** ^1^Zhejiang Provincial Center for Disease Control and Prevention, Hangzhou, China; ^2^Key Laboratory of Public Health Detection and Etiological Research of Zhejiang Province, Hangzhou, China; ^3^National HIV/AIDS Reference Laboratory, National Center for AIDS/STD Control and Prevention, Chinese Center for Disease Control and Prevention, Beijing, China; ^4^Fuyang District Center for Disease Control and Prevention, Hangzhou, China; ^5^Minhang Hospital, Fudan University, Shanghai, China; ^6^Yangpu Hospital, Tongji University, Shanghai, China; ^7^Institute of Laboratory Animal Sciences, Chinese Academy of Medical Sciences and Peking Union Medical College, National Human Diseases Animal Model Resource Center, Beijing, China

**Keywords:** hepatitis C virus, RNA, performance assessment, quantitative, serum panel

## Abstract

**Introduction:**

Accurate, specific, and sensitive detection and quantification of hepatitis C virus (HCV) RNA are critical for diagnosing and managing HCV infections. This study evaluated and compared the performance of four commercially available HCV RNA quantification reagents using standardized serum panels, providing evidence-based insights for clinical applications.

**Methods:**

Performance metrics, including analytical sensitivity, specificity, limit of detection (LOD), precision, genotype inclusivity (GT 1–6), and linearity, were assessed using seven distinct serum panels: basic, analytical specificity, seroconversion, analytical sensitivity, precision, genotype qualification, and linearity panels.

**Results:**

All reagents demonstrated 100% analytical sensitivity and specificity (95% CI: 79.95–100), with no cross-reactivity to common interfering substances or viruses. LOD values for reagents A, B, C, and D were 25, 50, 50, and 50 IU/ml, respectively. Intra- and inter-assay coefficients of variation (CVs) for HCV genotypes 1–6 ranged from 1.48 to 4.37% and 1.74 to 4.84%, respectively. Strong linear correlations (R^2^ > 0.95) were observed between measured and expected HCV RNA levels across all reagents.

**Conclusion:**

These reagents exhibit high sensitivity, specificity, precision, and accuracy for HCV genotypes 1–6, with a wide linear range, making them suitable for clinical diagnosis and monitoring of HCV infections.

## Introduction

Chronic hepatitis C (CHC) is a global health challenge caused by the hepatitis C virus (HCV), a single-stranded, positive-sense RNA virus belonging to the *Flaviviridae* family, characterized by an insidious onset and atypical clinical symptoms (Mohd Hanafiah et al., 2013). An estimated 71 million individuals worldwide are chronically infected with HCV, resulting in approximately 400,000 annual deaths from complications such as cirrhosis and hepatocellular carcinoma. If left untreated, most CHC patients may progress to liver fibrosis, compensated and decompensated cirrhosis, ultimately leading to hepatocellular carcinoma (HCC) and end-stage liver disease (ESLD)(Chen and Morgan, 2006). Although direct-acting antivirals (DAAs) have made it possible to cure CHC, many HCV-infected individuals remain undiagnosed, particularly in resource-limited settings (Collaborators, 2017; Xie et al., 2019; World Health Organization, 2021). In China, the burden of HCV infection is significant, with a seroprevalence of anti-HCV antibodies ranging from 0.43 to 0.6% in the general population, equating to 6–10 million infected individuals (Mei and Lu, 2021). China’s HCV elimination efforts encounter barriers related to testing access, treatment costs, and public awareness. Financial obstacles to HCV care persist despite insurance coverage for direct-acting antivirals (DAAs). Strategic cost-reduction measures—including centralized price negotiations, bulk procurement, and targeted subsidies—along with expanded insurance coverage for diagnostic testing and treatment monitoring are critical to enhancing equitable access for all patients. To achieve the World Health Organization (WHO) goal of eliminating HCV as a major global public health threat by 2030 (World Health Organization, 2016), it is essential to rely on prompt and accurate laboratory diagnostic methods and effective, feasible testing strategies (Poljak, 2020; Capraru and Feld, 2021; Roger et al., 2021).

The HCV genome is approximately 9.6 kilobases long and exhibits extensive genomic diversity, classified into 8 major genotypes and 90 subtypes, with distinct global distributions that influence clinical management and diagnostic challenges (Arca-Lafuente et al., 2024). Genotype 1 (GT1), which accounts for 66% of global infections, predominates in North America and Europe, while GT3 is prevalent in parts of Asia and Africa, often associated with accelerated liver fibrosis. In China, GT1b and GT2a are the most common subtypes; however, emerging subtypes (GT3 and GT6) in southern regions necessitate genotype-inclusive diagnostic assays to prevent detection failures (Huang et al., 2018; Mei and Lu, 2021; Li et al., 2025). This genetic heterogeneity presents challenges for diagnostic accuracy, as assays must encompass diverse genotypes to avoid missed infections.

The diagnosis of HCV infection relies heavily on clinical laboratory tests, including HCV antibody detection and nucleic acid testing (NAT). Anti-HCV antibody detection is generally regarded as a serological screening test for HCV infection (World Health Organization, 2018). It is relatively simple and cost-effective; however, due to the long “window period,” it poses a significant challenge in detecting patients during the acute phase of HCV infection and cannot differentiate between active HCV infections and spontaneous resolution (Austria and Wu, 2018; Seo et al., 2020). Accurate HCV RNA detection, as a supplementary test, plays a crucial role in the diagnosis, treatment, and management of HCV infection. On the one hand, HCV RNA detection exhibits high sensitivity and specificity in the diagnosis of HCV infection and can clarify the infection status. On the other hand, it can also monitor and evaluate the response to antiviral treatment and guide treatment decisions for HCV-infected patients (Martinello et al., 2018). Currently, several technologies have been developed for HCV RNA detection, such as branch DNA assay, real-time quantitative polymerase chain reaction coupled with reverse transcription (RT-qPCR), reverse transcription loop-mediated isothermal amplification (RT-LAMP) assays (Pauly et al., 2023), recombinase polymerase amplification (RPA)(Chia et al., 2022), and CRISPR/Cas-based diagnostic systems (Bahrulolum et al., 2023). RT-qPCR is widely considered the gold standard for HCV RNA detection, offering high sensitivity, with a detection limit (LOD) typically ranging from 5 to 50 IU/ml. The specificity is also extremely high, often exceeding 99%. For instance, the Roche COBAS AmpliPrep/COBAS TaqMan HCV Quantitative Test, v2.0, has an LOD of around 15 IU/ml and demonstrates excellent performance in accurately detecting HCV RNA (Huang et al., 2016).

In China, there is a wide availability of commercial HCV RNA quantification reagents, including internationally marketed assays from Roche, Siemens, Abbott, and Cepheid, as well as 17 domestically produced HCV RNA reagents currently registered with the National Medical Products Administration (NMPA) for clinical use. Furthermore, all domestically approved reagents utilize quantitative fluorescence-probing PCR technology. However, few independent performance evaluations of these products have been publicly reported, and different manufacturers may exhibit varying product performances, such as LOD and linear ranges. Thus, there is an urgent need to guide the implementation of accurate tests in a diagnostics market flooded with new tests (Chen et al., 2021). Herein, four HCV RNA quantification assays that apply real-time PCR technology with the PCR-fluorescence probing method commonly used in the market were evaluated and compared for performance using multiple serum panels in this study.

## Materials and methods

### Samples and reagents

The specimens in this study are sourced from two validated origins: commercial serum panels (SeraCare Life Sciences) and post-testing residual samples collected by the Chinese Center for Disease Control and Prevention from intravenous drug users and blood donors from routine HIV, HBV, and HCV testing. To address the limited sample size in commercial serum panels, specimens will be diluted in a matrix explicitly confirmed negative for all three pathogens through combined serological and nucleic acid testing (NAT).

Additionally, all samples underwent HCV antibody testing using ELISA reagents (Murex, DiaSorin; Wantai Biopharm) and HCV RNA quantification using COBAS® (Roche) assays. This study was approved by the Ethics Committee of National Center for AIDS/STD Prevention and Control, Chinese Center for Disease Control and Prevention (Number #X150619379).

Considering the types of HCV nucleic acid detection reagents commonly utilized in medical institutions in China, domestic brands with significant market share, and their registration and approval by the NMPA, four commercially available HCV RNA quantification reagents employing real-time fluorescent quantitative PCR technology were selected for the performance evaluation in this study. The Hepatitis C Virus (HCV) RNA Test Reagents (PCR-Fluorescent Probing) produced by Wantai BioPharm, Daan Gene, Beijing NaGene Diagnosis, and Kehua Bio-Engineering were designated as A, B, C, and D, respectively. The details of the reagents are summarized in Table 1.

**Table 1:** Details of selected HCV RNA quantification reagents.

Reagents	Nucleic acid extraction method	Nucleic acid amplification system	Specification/T	Sample Processing volume (μl)	Linear Range (IU/ml)	LOD (IU/ml)
A	Magnetic bead	Mx3000P, Bio-Rad CFX96, ABI 7500	48	500	50 ~ 10^8^	25
B	Acid extract method	ABI 7300, ABI 7500, Light Cycler 480	48	200	50 ~ 10^8^	20
C	Magnetic bead	SLAN-96P, Mx3000P, Mx3005P, ABI 7500	48	100	50 ~ 10^8^	15
D	Acid extract method	ABI 7300, ABI 7500, Mx3005P, Light Cycler 480	32	200	10^3^ ~ 10^7^	50

### Evaluation procedures

The project was conducted following the “Protocol for the laboratory evaluation of HCV molecular assays” and “Guidance on test method validation for *in vitro* diagnostic medical devices” issued by WHO. Four selected HCV RNA quantification reagents were evaluated using the HCV RNA basic panel, HCV analytical specificity panel, HCV seroconversion panel, HCV analytical sensitivity panel, HCV RNA precision serum panel, HCV RNA genotype qualification panel, and HCV RNA linearity panel, respectively. Each stock specimen from all HCV RNA serum panels was tested in parallel with these four reagents, following the manufacturer’s instructions. Subsequently, operations and data analysis were conducted under blinded or double-blinded conditions (Chen et al., 2021).

### Construction of HCV serum panels for performance assessment

The HCV RNA basic serum panel comprised 40 serum samples to evaluate the analytical sensitivity and specificity of selected HCV RNA reagents, including 20 HCV RNA-positive and 20 HCV RNA-negative samples.

The HCV analytical specificity panel was employed to assess the analytical specificity of the reagents. This panel contained 40 serum samples, identified as negative for HCV antibodies and HCV RNA but positive for HBV or HIV. Additionally, several samples exhibiting hemolysis, lipemia, or other interference factors were included.

The HCV seroconversion panel was utilized to evaluate the sensitivity of the reagents for early detection. Furthermore, the HCV seroconversion panel PHV928 (SeraCare Life Sciences) in this study consisted of 9 vials (1.0 ml per vial) of serum samples. Given the limited sample size, each specimen was diluted at 1: 9 with normal human serum that is negative for HIV, HBV, and HCV.

The HCV analytical sensitivity panel was employed to assess the LOD of the HCV reagents. The samples in this panel were prepared by diluting the WHO International Standard for HCV RNA (2.34 × 10^5^ IU/ml), resulting in concentrations of 100, 50, 25, 12.5, 5, and 0 IU/ml. Each concentration sample was tested in 20 replicates using the selected reagents.

The HCV RNA precision panel was primarily used to assess the precision and reproducibility of the HCV RNA quantitative reagents for detecting different sample levels. Moreover, the HCV precision panel in this study consisted of two samples with the following concentrations: 10^5^ and 10^4^ IU/ml, respectively. Each specimen was tested in quadruplicate for 5 days with selected HCV RNA quantitative reagents. Finally, coefficients of variation (CVs) for repeatability and precision were calculated.

The HCV RNA genotype qualification panel was utilized to assess the capability of HCV RNA reagents in detecting various HCV genotypes. The commercial HCV genotype qualification panel (No. 2400-0182; SeraCare Life Sciences) employed in this study comprised 9 specimens, including genotypes HCV 1a, 1b, 2a, 2b, 3a, 4a, 5a, 6, and HCV RNA negative plasma.

The HCV RNA linearity panel was employed to assess the dynamic linear range of the HCV RNA quantitative reagents. The commercial HCV RNA Linearity panel (No. PHW805; SeraCare Life Sciences) used in this study included seven positive samples and one negative sample. Furthermore, 7 positive samples were prepared by serial 10-fold dilutions of HCV RNA-positive positive with negative samples. Subsequently, each sample of this panel was tested with four selected reagents and compared to the expected results.

### Statistical analysis

Data analysis was conducted using Excel 2021 and GraphPad 8.0 software. The results of HCV RNA quantitative were converted into logarithmic values (log IU/ml), and the 95% confidence interval (CI) was calculated using a Bayesian assay. Linear regression was employed to describe the relationship between the results of different reagents in the HCV linearity panel and the expected outcomes. The LOD of the reagents was determined as the concentration at which the positive rate was 95%.

## Results

### HCV RNA basic serum panel

The analytical sensitivity and specificity of the four HCV RNA reagents evaluated were assessed using the basic serum panel. The results from this study showed that the analytical sensitivity of the included reagents was 100% (95% CI: 79.95–100), and the analytical specificity was also 100% (95% CI, 79.95–100), with no false positive or false negative results.

### HCV analytical specificity panel

To further assess the analytical specificity of the four reagents, we constructed an HCV analytical specificity panel using samples that commonly exhibit cross-reactivity with other viral infections, such as HIV and HBV. The results indicated that the 40 samples with interference factors detected by HCV RNA quantitative reagents were negative, confirming that the analytical specificity of these reagents was 100% (95% CI: 89.09–100.00).

### HCV seroconversion panel

The sensitivity of HCV RNA reagents in early detection in this study was evaluated using a commercially available HCV seroconversion panel, PHV928. Results from the included HCV RNA regents were compared with the expected outcomes, revealing that no reagent could detect viral nucleic acid at the early stage of HCV infection. As the viral load increased, the reagents demonstrated reduced sensitivity in early detection compared to the reference assay, particularly reagent C (Table 2).

**Table 2:** HCV seroconversion panel with different HCV RNA reagents.

Panel member	Reference HCV RNA results (IU/ml)^1^	Expected HCV RNA results (IU/ml)^2^	Reagent A	Reagent B	Reagent C	Reagent D
1	BLD^3^	BLD^3^	TND^3^	TND^3^	TND^3^	TND^3^
2	BLD^3^	BLD^3^	TND^3^	TND^3^	TND^3^	TND^3^
3	5.9E+01	5.9	TND^3^	TND^3^	TND^3^	TND^3^
4	1.1E+05	1.1E+04	3.76E+03	1.22E+04	8.48E+01	2.23E+03
5	1.6E+06	1.6E+05	4.41E+04	8.03E+04	1.58E+02	3.97E+04
6	4.2E+06	4.2E+05	9.14E+04	1.68E+05	2.93E+02	5.23E+04
7	1.5E+07	1.5E+06	3.61E+05	1.24E+06	2.96E+03	1.98E+05
8	5.0E+06	5.0E+05	6.25E+04	1.07E+06	7.86E+02	3.44E+04
9	9.7E+06	9.7E+05	2.43E+05	1.13E+06	9.86E+02	9.73E+04

1 Reference results were tested by Roche COBAS®AmpliPrep/COBAS® TaqMan® HCV Quantitative Test, v2.0 with undiluted samples.

2 Expected results were calculated by diluting to one-tenth of its original concentration.

3 BLD, below limit of detection; TND, target not detected.

### HCV analytical sensitivity panel

The analytical sensitivity was assessed using the HCV analytical sensitivity panel, with samples at each concentration tested 20 times (Table 3). The results indicated that all reagents were detected in the samples at 100 IU/ml. The positive detection rates for reagents A, B, C, and D were 100, 95, 100, and 100%, respectively, at a sample concentration of 50 IU/ml. Consequently, the LODs for reagents A, B, C, and D were estimated to be 25, 50, 50, and 50 IU/ml, respectively, aligning with the minimum detection limits stated by the manufacturers.

**Table 3:** HCV analytical sensitivity panel.

Expected HCV RNA concentration (IU/ml)	Replicates	Positive detection rates			
		Reagent A	Reagent B	Reagent C	Reagent D
100	20	100% (20/20)	100% (20/20)	100% (20/20)	100% (20/20)
50	20	100% (20/20)	95% (19/20)	100% (20/20)	100% (20/20)
25	20	100% (20/20)	90% (18/20)	85% (17/20)	90% (18/20)
12.5	20	85% (17/20)	75% (15/20)	60% (12/20)	50% (10/20)
5	20	45% (11/20)	70% (14/20)	20% (4/20)	25% (5/20)
0	20	0% (0/20)	0% (0/20)	0% (0/20)	0% (0/20)

### HCV precision serum panel

The intra-assay and inter-assay coefficients of variation (CVs) for HCV RNA quantification were utilized to evaluate the precision and repeatability of the HCV RNA quantification reagents. The intra- and inter-assay CVs for all reagents ranged from 1.48 to 4.37% and 1.74 to 4.84%, respectively, both remaining below 5%, which indicates that these four reagents exhibit good precision and repeatability (Table 4).

**Table 4:** HCV precision serum panel.

Reagents	Samples	$\bar{x}$	Standard Deviation	CV%	Inter-assay CV%	Intra-assay CV%
Reagent A	P1	5.54	0.17	3.08	0.86	3.01
	P2	4.54	0.12	2.67	1.90	2.22
Reagent B	P1	5.96	0.26	4.28	2.42	3.85
	P2	4.34	0.21	4.84	2.27	4.37
Reagent C	P1	3.78	0.18	4.42	3.82	3.29
	P2	2.55	0.13	4.81	3.66	3.89
Reagent D	P1	5.23	0.09	1.74	1.16	1.48
	P2	4.12	0.17	4.12	2.57	3.60

### HCV RNA genotype qualification panel

The capability of detecting various HCV genotypes was evaluated using a commercially available HCV RNA genotype qualification panel. The results demonstrated that samples with different HCV genotypes in this panel could be identified by these four reagents (Table 5). This indicates that these four reagents can accurately quantify the viral load of most HCV genotypes.

**Table 5:** HCV RNA genotype qualification panel.

Panel Member	HCV Genotype	Reference Results (IU/ml)	A (IU/ml)	B (IU/ml)	C (IU/ml)	D (IU/ml)
01	1a	7.70E+04	1.49E+03	2.36E+03	6.92E+02	7.23E+02
02	1b	3.60E+04	2.95E+03	9.18E+03	7.84E+01	1.85E+03
03	2a/2c	1.00E+05	1.11E+04	1.72E+04	3.11E+02	7.74E+03
04	2b	2.30E+04	3.90E+02	7.86E+02	1.81E+02	1.57E+02
05	3	2.10E+04	2.78E+03	2.85E+03	2.28E+02	1.04E+03
06	4	2.90E+04	3.34E+03	3.54E+03	1.18E+03	1.19E+03
07	5	1.10E+05	1.42E+04	2.69E+04	1.14E+03	5.00E+03
08	6	5.30E+04	2.82E+04	1.25E+04	3.02E+03	5.39E+03
09	NA	TND	TND	TND	TND	TND

TND, target not detected.

### HCV RNA linearity panel

**Figure 1:**
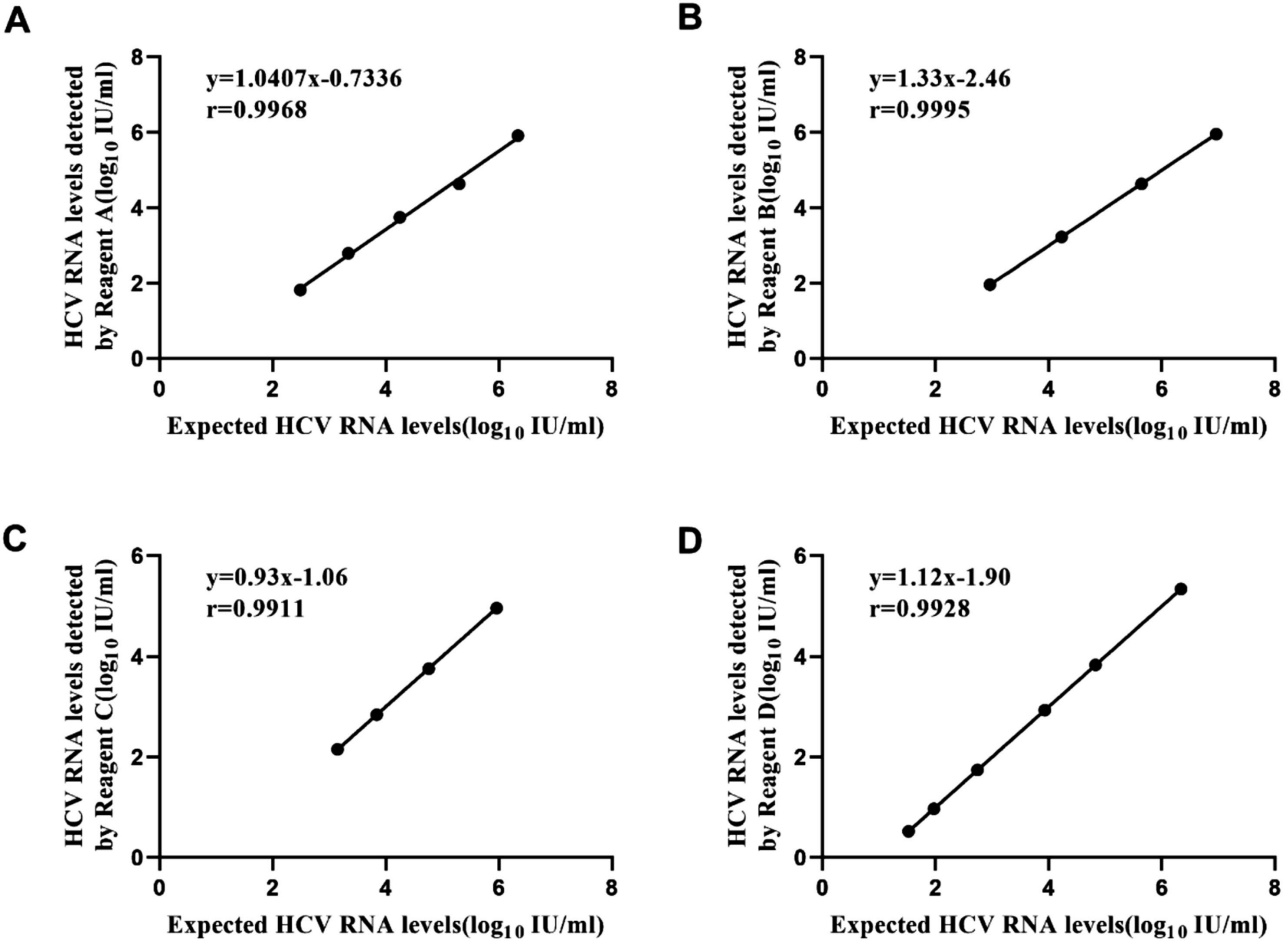
Linear regression of logarithmic results and expected values of the HCV linearity panel with four reagents. Reagent A (slope = 1.0407) and Reagent C (slope = 0.934) demonstrated regression slopes close to unity, indicating minimal proportional bias. By contrast, Reagent B (slope = 1.3332) and Reagent D (slope = 1.1204) exhibited steeper regression slopes, suggesting their viral load measurements systematically overestimated values relative to the reference standard. **(A)** Performance of Reagent A. **(B)** Performance of Reagent B. **(C)** Performance of Reagent C. **(D)** Performance of Reagent D.

The correlation between HCV RNA levels detected by four HCV RNA quantification assays and the expected results was evaluated using the HCV RNA linearity panel. As illustrated in Figure 1, a significant relationship was observed between the HCV RNA levels detected by regents A, B, C, D, and the expected HCV RNA levels (r_A_ = 0.998, P_A_ < 0.01, regression equation: y = 1.0407x − 0.7336; r_B_ = 1.000, P_B_ < 0.01, regression equation: y = 1.3332x − 2.4629; r_C_ = 0.996, P_C_ < 0.01, regression equation: y = 0.934x − 1.0603; r_D_ = 0.996, P_D_ < 0.01, regression equation: y = 1.1204x − 1.9039). All tested reagents demonstrated remarkable linear correlation between detected and expected HCV RNA levels, as evidenced by near-perfect Pearson’s r coefficients ranging from 0.996 to 1.000 (*p* < 0.01 for all comparisons).

## Discussion

Accurate, specific, and sensitive detection and quantification of HCV RNA levels are essential for the diagnosis and management of HCV infection (Yao et al., 2018). Currently, numerous HCV RNA quantification reagents have been registered with the NMPA, most of which utilize real-time quantitative PCR technology to detect HCV RNA levels, offering high sensitivity and specificity. In this study, we systematically evaluated the analytical sensitivity and specificity of four commonly used HCV RNA quantification reagents in China for the first time. We found that 20 HCV RNA-positive and 20 HCV RNA-negative samples in the basic panel could be accurately detected by these four reagents. This indicates that the four reagents assessed in this study possess high analytical sensitivity and specificity. Additionally, we calculated the intra-assay and inter-assay coefficients of variation for the four reagents when detecting samples with various HCV RNA levels, revealing that both intra-assay and inter-assay CV were below 5%. These assays demonstrated excellent precision and repeatability, aligning with the manufacturer’s claims (He et al., 2020).

Furthermore, patients with CHC exhibit a wide range of HCV RNA levels, necessitating sensitive HCV RNA quantification assays to monitor treatment efficacy in HCV-infected individuals (Visco-Comandini et al., 2018; Mücke et al., 2019). Consequently, HCV RNA quantitative assays must possess large dynamic ranges and low LOD to yield reliable results (World Health Organization, 2021). In this study, significant correlations were observed between the four HCV RNA quantitative reagents and the expected concentrations detected by the COBAS AmpliPrep/COBAS TaqMan HCV Quantitative Test. According to the analytical sensitivity results, reagents A, B, C, and D achieved the LOD ≤ 50 IU/ml required for registration by the NAMP. However, the LOD of these four reagents differed from that of the COBAS AmpliPrep/COBAS TaqMan HCV Test (LOD = 15 IU/ml) and Abbott Real-time HCV Assay (LOD = 12 IU/ml), which may be attributed to variations in the recommended sample processing volumes (Thodou et al., 2020), such as 500 μl for reagent A, 200 μl for reagent B, and 700 μl for the COBAS assay. Among the four HCV RNA detection reagents evaluated in this study, the limit of detection (LOD) ranged from 15 IU/ml (demonstrating superior sensitivity) to 50 IU/ml. Notably, all reagents consistently detected HCV RNA in 100% of repeated tests (20/20 replicates) at both 50 and 100 IU/ml concentrations. These results underscore the reagents’ exceptional analytical performance, characterized by high sensitivity (≤50 IU/ml, aligning with internationally accepted diagnostic thresholds) and robust reproducibility across low viral load ranges. In this context, the four commercially available HCV RNA assays included in this study are excellent and capable of detecting and quantifying less than 50 IU/ml or less, thereby meeting routine clinical requirements. It is essential to effectively monitor lower viral titers during antiviral therapy in HCV-infected patients in clinical practice (Chan et al., 2020; Elabd et al., 2021).

Moreover, HCV GT is utilized to guide therapeutic decisions and preventive strategies (Martinez and Franco, 2020; Hu Hh et al., 2021). We found that samples with HCV GT 1a, 1b, 2a, 2b, 3a, 4a, 5a, and 6 in the HCV RNA genotype identification panel can be effectively detected by the four reagents, meeting clinical requirements. Additionally, the anti-interference capability of these four HCV RNA reagents was assessed using common interfering substances. It appeared that these reagents exhibited no cross-reactivity and demonstrated excellent analytical specificity, consistent with previous studies (Vermehren et al., 2008). Subsequently, the sensitivity of HCV RNA reagents for early detection was evaluated using a commercially available HCV seroconversion panel. Compared to the reference results, the early detection capabilities of the four reagents were relatively low.

The cost-effectiveness of HCV detection reagents is a crucial factor in achieving universal access to diagnosis, particularly in resource-limited settings. Real-time PCR assays, such as Roche’s COBAS AmpliPrep/COBAS TaqMan HCV Test v2.0, provide superior sensitivity and genotype-independent performance but come with high costs, limiting their application in low-income regions. While DAAs are now reimbursed under China’s national medical insurance, diagnostic costs are not universally covered. A study conducted between 2016 and 2018 across 76 hospitals in China analyzed patients with HCV infection and revealed that 48.4% tested positive for anti-HCV antibodies. Among these, only 34.9% were confirmed to have detectable HCV RNA, indicating a substantial gap in diagnostic follow-up, highlighting significant challenges in translating screening results into clinical care (Mei and Lu, 2021).

Our study has its limitations. First, we constructed various serum panels by selecting special samples from high-risk HCV infections based on their background information. Second, some samples in serum panels, which had a limited sample size, were diluted with normal human serum that tested negative for HIV, HBV, and HCV. Third, while there are 17 HCV RNA quantitative reagents approved by NMPA in China, not all RT-PCR reagents available on the market could be included, only four commonly available reagents were selected for performance evaluation.

In China, with increased investments in research and development for diagnostic reagents, the quality of *in vitro* diagnostic reagents has been continuously improving, demonstrating good correlation with international standards, such as Daan Gene and Sansure assays (Huang et al., 2016; He et al., 2020). The four HCV RNA quantification reagents evaluated in this study all exhibit excellent characteristics, including high analytical sensitivity, analytical specificity, good precision, low detection limits, wide linear range, and good agreement with the COBAS TaqMan 2.0 assay. This suggests that the HCV RNA reagents in China provide cost-effective alternatives for the diagnosis and monitoring of HCV infection in the clinical setting. In the future, decentralized tools like rapid diagnostic tests (RDTs) and mobile clinics could expand screening in rural areas, while point-of-care RNA technologies enable faster diagnosis. By integrating automated domestic RT-PCR platforms, improving reimbursement, and implementing micro-elimination strategies targeting high-risk groups, China can accelerate progress toward HCV elimination by 2030.

## Data Availability

The raw data supporting the conclusions of this article will be made available by the authors, without undue reservation.
